# Cardiac Amyloidosis: Clinical Features, Pathogenesis, Diagnosis, and Treatment

**DOI:** 10.5146/tjpath.2023.12923

**Published:** 2024-01-22

**Authors:** Asuman Argon, Deniz Nart, Funda Yılmaz Barbet

**Affiliations:** Department of Pathology, Health Sciences University, Izmir Faculty of Medicine, Izmir, Turkey; Ege University, Faculty of Medicine, Izmir, Turkey

**Keywords:** Amyloid, Cardiac amyloidosis, Transthyretin, Hereditary amyloidosis, Senile amyloidosis

## Abstract

Cardiac amyloidosis is a type of amyloidosis that deserves special attention as organ involvement significantly worsens the prognosis. Cardiac amyloidosis can be grouped under three main headings: immunoglobulin light chain (AL) amyloidosis that is dependent on amyloidogenic monoclonal light chain production; hereditary Transthyretin (TTR) amyloidosis that results from accumulation of mutated TTR; and wild-type (non-hereditary) TTR amyloidosis formerly known as senile amyloidosis. Although all three types cause morbidity and mortality due to severe heart failure when untreated, they contain differences in their pathogenesis, clinical findings, and treatment. In this article, the clinical features, pathogenesis, diagnosis, and treatment methods of cardiac amyloidosis will be explained with an overview, and an awareness will be raised in the diagnosis of this disease.

## DEFINITION and HISTORY

Amyloidoses are a group of diseases that exhibit heterogeneity. Their common feature is that they have accumulations of abnormal proteins that result in tissue and organ damage. Historically, the term “amyloid” (a normal amylous component in plants) was first suggested in 1838 by German botanist Matthias Schleiden (1). He named it “corpora amylacea”, describing the small round structures in the nervous system that give a characteristic color reaction (brown to blue) with sulfuric acid andiodine, which is typical for starch (2). In 1861, Dr. T. Grainger Stewart described the deposits in the kidney (in Bright’s disease) as a “waxy or amyloid form” (3). Pathologist John W. Budd was the first scientist to report one of the cardiac amyloidosis (CA) cases in the literature, saying, “Although primary amyloid disease of the heart is very rare, the cases reported in the literature show that hyaline material can accumulate in the epicardium, myocardium, endocardium, valves or walls of adjacent blood vessels” (4).

## INCIDENCE

The reported incidence for CA is 18-55 per 100,000 person-years. In fact, its prevalence is difficult to determine precisely, because the disease is often overlooked as it often presents with non-specific symptoms (5). A population-based autopsy study suggests that 25% of people aged 80-85 years have cardiac amyloid deposition (6). The UK National Amyloidosis Center database report, which includes data on 11006 patients between 1997 and 2019, reports that the number of patients suffering from amyloidosis increased by 670% from 1987-1999 to 2010-2019 (7). Again, the same report emphasizes that the incidence of CA, which was less than 3% of all cases in the 1987-2009 period, increased to 14% in the 2010-2015 period and to 25% in the last 4 years. It is conceivable that this increased incidence may be the result of increased awareness of amyloidosis and diagnostic cardiac imaging.

## CLINICAL FEATURES

The disease usually first presents with shortness of breath secondary to exertion, which is partly rapidly progressing and results in peripheral edema and/or ascites. Left ventricular diastolic dysfunction results in dyspnea. However, significant hardening of the auricles likely contributes to exertional dyspnea. The first signs of the disease include cardiac arrhythmias (atrial/ventricular) and heart block of varying degrees (8,9). Deposits in the atrium cause dysfunction, and thrombi can form even if the heart is operating in sinus rhythm. This causes thromboembolism to be seen as an early sign of the disease. If the clinician is not aware of the phenomenon of left atrial systolic dysfunction that is the source of neurological or systemic embolism, the disease can easily be overlooked (10). Electrical conduction disturbances in the heart due to amyloid deposition, embolic events, and syncope are some of the reasons for hospital admission for affected individuals. However, patients often come to the health institution with cardiogenic shock. In recent years, it can be said that awareness of cardiac dysfunction caused by amyloidosis has increased (11). In addition, it is reported that toxic infiltrative cardiomyopathy from cardiac amyloidosis is much more common than previously believed and is an underrecognized cause of diastolic heart failure in particular (12).

## PATHOGENESIS

Among the amyloidoses, CA has a special importance because organ involvement significantly worsens the prognosis. Cardiac amyloidoses are grouped under three headings: AL amyloidosis due to amyloidogenic monoclonal light chain production of a plasma cell clone; hereditary TTR amyloidosis (ATTRv) caused by accumulation of mutated Transthyretin (TTR); and wild-type (non-hereditary) TTR amyloidosis (ATTRwt) (13). Rarely, cardiac involvement can be seen in secondary amyloidosis (14).

**AL amyloidosis **is a multi-organ disease, although involvement of one organ is usually predominant. The kidney is the first organ to be affected, and it manifests itself with nephrotic syndrome. The second most affected organ is the heart. There is a slight male dominance. Although the disease can be seen at any age from the fourth decade, it often occurs after the fifth decade (14). AL amyloidosis is known to be the most severe of CAs. If the disease is not treated, the patient is expected to die within about 6 months after the onset of heart failure (15). Although there is greater accumulation in the left ventricle in TTR amyloidosis, heart failure has been shown to be more severe in AL amyloidosis than in TTR amyloidosis (14,16). Studies focusing on the pathogenesis of cardiac AL amyloidosis have shown that amyloidogenic light chains cause an increase in reactive oxygen products (ROP) and upregulation of heme oxygenase in rat cardiac muscle cells. Unfortunately, this process results in the deterioration of contraction and relaxation (14,17). The initial response to amyloid deposition appears as lysosomal dysfunction. This leads to generation of highly ROP, functional loss in cells, impaired calcium homeostasis, and cell death, with disruption of autophagy (14,18). The unbranched amyloid fibrils not only consist of precursor protein units, but also contain serum amyloid P and proteoglycans. Evidence has shown that amyloid can be reabsorbed, albeit slowly, after fibrillogenesis is stopped but generally the other proteins it contains are extremely resistant to degradation (14,19,20). AL CA studies suggest that AL amyloidosis may have infiltrative as well as toxic effects (21). In the light of the evidence, it would be more accurate to consider AL cardiac amyloidosis both as an infiltrative heart disease and as an infiltrative toxic cardiomyopathy.

**Transthyretin amyloidosis: **The main function of Transthyretin (prealbumin) produced by the liver is to transport thyroxine and retinol. The gene encoding the protein is located on chromosome 18. Although it can acquire monomeric amyloidogenic properties in conditions such as genetic damage and aging, it is a homotetramer in its normal state. It has been shown that monomeric amyloidogenic intermediates can subsequently spontaneously revert to amyloid fibrils (22). Point mutations that destabilize the tetramer are involved in hereditary amyloidogenesis (23). More than 100 amyloidogenic mutations have been reported in TTR. However, the most common cause of familial amyloid cardiomyopathy is the isoleucine mutation at position 122, which is also seen in 3.9% of the Afro-Caribbean population (V122I) (24). Caucasian variant mutations have also been identified: Leu111Met (Denmark), Ile68Leu (Italy), and Thr60Ala (Appalachian and Irish regions) (25).

The mechanism of heart failure in individuals carrying the V1221 allele has not been clearly elucidated. However, it is suggested that individuals carrying this allele have an increased risk of heart failure and death, particularly at the age of 60 to 65 years (26). Connors et al.’s study showed that ATTRv patients with the V122I mutation were older, had more pronounced ventricular hypertrophy, had lower left ventricular ejection fractions, and had more atrial dilatation on echocardiography (ECO) than Black Americans with AL amyloidosis. However, despite these findings, the patients’ symptoms are less severe (27,28). An isolated cardiomyopathy is often an expected finding in individuals with the V122I mutation. It is not surprising that cardiac involvement and familial amyloid polyneuropathy are more common in individuals with TTR gene mutations, which are endemic in Europe and Asia. (28). Applications to healthcare institutions with non-cardiac symptoms such as purpura, easy bruising, carpal tunnel syndrome, and peripheral polyneuropathy are substantial (8). When examining the mechanism of cardiac damage, as mentioned above, it is well known that light chains have direct toxicity to myocytes from those affected by AL amyloidosis (21). However, it has been shown that mutant transthyretin fibrils can trigger different mechanisms that cause damage in cardiac muscle cells. These mechanisms include triggering the proinflammatory cascade mainly by NF-kB activation, disruption of calcium metabolism, and downregulation of proteosomal activity (28,29).

**Figure 1:**
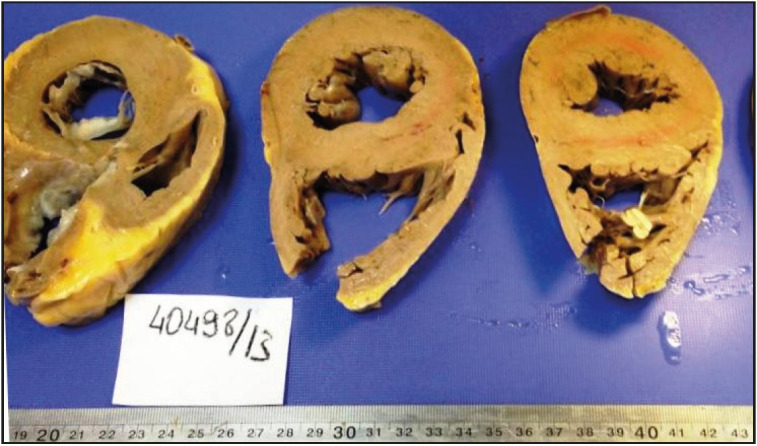
Ventricular thickness increase due to amyloid deposition in the explant material of the patient who underwent heart transplantation due to ATTRv.

**Transthyretin-associated nonhereditary amyloidosis (ATTRwt):** The main reason ATTRwt is called senile systemic amyloidosis (SSA) is that the disease often begins after the age of 70. Similar to AL amyloidosis, it has a strong male predominance (6,20). The prevalence of ATTRwt amyloidosis is not known precisely because the diagnosis cannot be made in many cases. Prolongation of life expectancy and the availability of modern diagnostic tools such as cardiovascular magnetic resonance imaging (CMR) and Technetium-99m-3,3-diphosphono-1,2-propanodicarboxylic acid (99m Tc-DPD) scintigraphy can be counted as the reason for the increasing prevalence today (7,10,20,30). ATTRwt type CA is thought to result from misfolding in the TTR due to advancing age (12). Oxidative modifications of proteins in aging-related proteogenesis and damage repair mechanisms are thought to contribute to the degradation and fibrillation of native TTR (31). Cardiac symptoms in ATTRwt patients usually present as shortness of breath, fatigue, and malaise, and these symptoms are generally not considered to be related to old age and further investigation is not performed. This is one of the most important reasons for the delay in diagnosis (6,8,9). Cardiac vascular amyloid deposition is a major cause of anginal chest pain, which can occur even in patients without obstructive coronary stenosis. Patients have heart failure but typically preserved ejection fraction (32). Studies suggest that mean left ventricular wall thickness is greater in ATTRwt than in ATTRv (33). Figure 1 shows the increase in ventricular thickness in the explant material of the patient who underwent cardiac transplantation due to ATTRv at Ege University (Figure 1). ATTRwt is expected to have a much better natural course than other amyloid cardiomyopathies (12,34). In one of the first studies, the median survival from admission with symptoms of heart failure was 60 months, compared to 5.4 months in patients with cardiomyopathy from AL amyloidosis (33).

The distinction between ATTRwt and ATTRv needs to be made carefully as the families of patients diagnosed with ATTRv should be given genetic counseling and their family members should be screened for this disease. Also, if the individual has a V122I mutation, closer monitoring is required as aggressive progression may occur. In addition, a confirmed diagnosis of ATTRv is essential for the patient to receive only treatments approved for ATTRv (35). Although some authors propose a diagnostic algorithm including NT pro-BNP level and age at diagnosis to distinguish senile ATTR cardiac amyloidosis from AL primary cardiac amyloidosis, it should not be forgotten that the definitive distinction can only be made with endomyocardial biopsy (EMB) (36,37).

## DIAGNOSIS

The first steps on the way to the correct diagnosis of cardiac amyloidosis is obtaining a detailed history, evaluating the symptoms, and clinically suspecting the disorder as a result of the examination. This suspicion is followed by laboratory studies and cardiac imaging.

### Scanning

International experts (ASNC/AHA/ASE/EANM/HFSA/ISA/SCMR/SNMMI) recommend the appropriate use of ECO, CMR, and radionuclide imaging in the diagnosis of cardiac amyloidosis and/or in the evaluation of patients with cardiac amyloidosis (38). ECO has the ability to offer clues to further testing, and CMR has the ability to show an infiltrative process. However, 99m technetium pyrophosphate scintigraphy allows for non-invasive diagnosis os CA, although the mechanism of attachment of radioactive material to amyloid deposits cannot be fully elucidated. In this respect, it is considered as an important milestone in the clinical diagnosis of cardiac amyloid (35,39). It has been reported that cardiac amyloidosis can be diagnosed by evaluating the patient together with non-cardiac biopsy, Tc-99m pyrophosphate scintigraphy and positron emission tomography data using molecules targeting myocardial amyloid uptake, as well as echocardiographic and CMR findings in cases where the disease cannot be proven with endomyocardial biopsy (38) (Table 1). In addition, it was suggested by the same authors that the need for invasive endomyocardial or extracardiac biopsy is eliminated if there are consistent echo or CMR findings in patients without monoclonal plasma cell increase, as well as findings consistent with ATTR cardiac amyloidosis on 99m Tc-PYP/DPD/HMDP scintigraphy.

**Table 1:** Diagnostic Criteria for Cardiac Amyloidosis (Dorbala, 2019)

**Diagnostic Criteria**	**Subtype**
Histology of Endomyocardial biopsy	
1. Endomyocardial biopsy showing birefringent amyloid deposition in apple green to polarized light in Congo red. Immunohistochemistry and/or mass spectrometry may be preferred for subtyping.	**AL, ATTR, other subtype**
Histology of Extracardiac biopsy	
1. The following conditions should be sought in the diagnosis of ATTR amyloidosis: a) Demonstration of ATTR amyloidosis on extracardiac biopsy, **and** b) Presence of typical cardiac imaging features described below	**ATTR**
2. The following conditions should be sought for the diagnosis of AL amyloidosis: a) Demonstration of AL amyloidosis on extracardiac biopsy, and b) Presence of typical cardiac imaging features described below c) Abnormality of age-adjusted NT pro BNP or abnormal Troponin T/I/HS Troponin levels; **after exclusion of all causes to explain the abnormality of these markers**	**AL**
Clinical Diagnosis of ATTR Cardiac Amyloidosis: 99m Tc-PYP/DPD/HMDP	
3. The diagnosis of ATTR amyloidosis can be made in the presence of the following conditions: a) Presence of Grade 2 or 3 myocardial involvement in 99m Tc-PYP/DPD/HMDP, **and** b) Demonstration of clonal plasma cell absence by serum FLCs and serum and urine immunofixation, **and** c) Presence of typical cardiac imaging features described below	**ATTR**
**Typical Imaging Features in the Diagnosis of Cardiac Amyloidosis**	
Typical cardiac echocardiography or CMR or PET features: All other diseases (including hypertension) that may produce the following imaging findings should be excluded	
1. Echocardiography • Left ventricular wall thickness >12mm • Relative apical preservation of global LS ratio (apical LS mean/combined mean+basal LS >1 mean) • ≥ Grade 2 diastolic dysfunction	**ATTR/AL**
2. Cardiac MRI a) LV wall thickness above the upper limit of normal for gender in unstable condition b) Overall ECV >0.40 c) Diffuse late gadolinium increase d) Abnormal gadolinium kinetics typical for amyloidosis, resetting myocardium before blood pool reset	**ATTR/AL**
3. PET: 18F-florbetapir or 18F-florbetaben PET a) Target - background (LV myocardium - blood pool) ratio >1.5 b) b. Retention index >0.030 min-1	**ATTR/AL**

### Laboratory

Patients with suspected CA after evaluation should first be evaluated for monoclonal gammopathy for AL-CA (40). Laboratory tests should include serum-free kappa and lambda light chains, as well as immune-fixed serum and urine protein electrophoresis (SPEP/UPEP with IFE). The sensitivity of serum plasma electrophoresis for AL amyloidosis is lower than that of serum IFE (~70% and >90%, respectively) (41). Troponin, BNP (brain natriuretic peptide) and NT-proBNP (N-terminal probrain natriuretic peptide) are other markers whose usefulness in the diagnosis and for predicting prognosis has been investigated. In AL-CA, increased production of monoclonal AL also activates the MAP kinase signaling pathway, increasing natriuretic peptide production. This results in elevated brain natriuretic peptide (BNP) and N-terminal proBNP level (42). However, the utility of these tests in the diagnosis of TTR-CA is very limited. Patients with ATTR amyloidosis often require EMB to confirm the diagnosis. When ATTR amyloidosis is detected, genetic testing for the TTR gene mutation should be performed. In addition, it has been reported that circulating retinol-binding protein 4 may be useful in identifying patients with ATTR-CA with V122I mutant heart failure (43). It should not be forgotten that a high troponin level can be used to predict a worse prognosis in both AL-CA and ATTR-CA, even if it is not in the diagnosis (37).

### Biopsy

**Figure 2:**
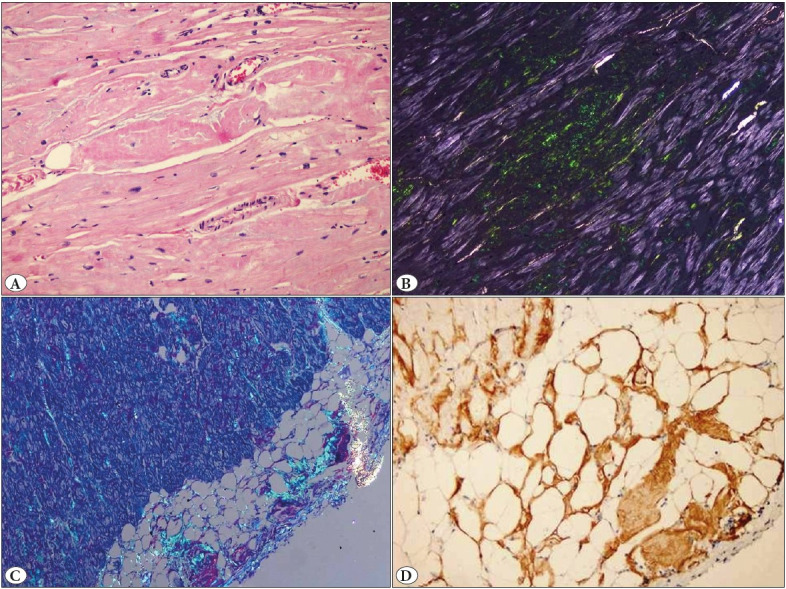
Histopathological features of ventricular muscle samples taken from the explant material of the patient who underwent heart transplantation due to ATTRv. **A**) Pericellular nodular deposits with hematoxylin-eosin stain, x10; **B**) Pericellular nodular amyloid deposits with apple green reflectors on Congo red staining, x10; **C**) Subendocardial and pericellular amyloid deposits with apple green reflectors on Congo red staining, x4: **D**) Positivity with immunohistochemical Transthyretin, x10.

In patients with suspected cardiac amyloidosis, scintigraphy or biopsy should be performed after monoclonal protein tests (44). While biopsy is valid for all forms of cardiac amyloidosis, noninvasive criteria are acceptable for ATTR only (45). In cases with monoclonal proteinemia, the accumulated amyloid form can be detected in biopsy taken from affected organ (such as endomyocardial, abdominal fat, bone marrow) (46). Because of its invasive nature, endomyocardial biopsy carries a small risk of complications, which can be serious. Its implementation requires technical expertise. Fat pad biopsy is less invasive and carries less risk, but its sensitivity in ATTR-CA is highly variable (47). All types of cardiac amyloidosis cause extracellular amyloid deposition and the accumulating form of amyloid is not expected to be distinguished by light microscopy. Although the type of amyloid deposition cannot be clearly differentiated from the deposition pattern, AL amyloidosis predominantly presents with pericellular, endocardial, and arterial and/or arteriolar deposits; nodular deposits are usually seen in ATTR (Figure 2) (48). Regardless of left ventricular wall thickness, the diagnosis of CA is confirmed when amyloid deposition is demonstrated on endomyocardial biopsy with Congo red (Figure 2). Once amyloid deposition is detected, the next step should be the classification of the amyloid fibril protein. The gold standard in classification is mass spectrometry, immunohistochemistry, or immunoelectron microscopy, which are routinely used in specialized centers (Figure 2) (49). In a study of 117 patients with amyloid, immunohistochemical analysis was reported to have 96% sensitivity and 100% specificity in identifying hereditary amyloidoses (50). In Ege University, where the first cardiac transplantation was performed in 1998, myocardial biopsy has been used for the diagnosis of cardiac diseases for approximately 25 years. In cases where clinical, laboratory data or histopathology arouses suspicion, amyloid accumulation in the tissue is investigated with histochemical congo red. When cardiac amyloidosis accumulation is detected, immunohistochemical subtyping is performed with Amyloid A, C4d, Fibrinogen, Pre Albumin/Transthyretin (TTR), Lambda, Lysozyme, and Kappa. Evaluation using these antibodies provides sensitivity and specificity rates similar to the study of Schönland et al. in detecting hereditary amyloidosis in our center.

## TREATMENT

### Medical Treatments

In patients with cardiac amyloid, the two ventricles are usually affected together; moreover, the left ventricle is greatly shrunk secondary to muscle hypertrophy. Therefore, durable left ventricular assist devices are often not a good option (51). Amyloid deposition, which causes damage to organs, is seen in both ATTR and AL amyloidosis, but the treatment regimens of these diseases are different (52). The main agent used in the treatment of ATTR is TTR silencing, which is involved in the synthesis of TTR in the liver, TTR stabilization that prevent misfolding by binding to the tetramer, and TTR disruption that ensures the clearance of amyloid from the organism (35). The TTR stabilizer tafamidis, which was approved in 2019, has taken its place in the treatment of amyloid cardiomyopathy (7,53). In addition, new treatments such as patisiran and inotersen, which reduce hepatic TTR production in hereditary ATTR amyloidosis, are also promising (7,54,55).

Since light chain toxicity causes cardiac damage in AL amyloidosis, correction of the relevant light chain should be the main goal (52). Autologous stem cell transplantation (ASCT) is among the emerging treatments for AL amyloidosis patients and has been shown to have very good long-term results. However, it has been reported that it may be an appropriate treatment option in a small proportion of patients with CA (56). The advent of effective anti-plasma cell therapies has changed the definition of hematological response from a complete response to a modified, strict, and absolutely relevant free light chain response. The agents used in anti-plasma cell therapy are mainly alkylating agents, immunomodulators, corticosteroids, and proteasome inhibitors (52).

### Orthotopic Cardiac Transplantation

Cardiac transplantation (CTx) may be considered in cardiac AL amyloidosis responding to light chain suppressive therapies and in cases with end-stage heart failure secondary to ATTR. In the past, cardiac amyloidosis, especially the AL type, was a contraindication for heart transplantation (HTx) because the disease is systemic and carries a high risk of death. However, effective therapies used today, including proteasome inhibitors, have made heart transplantation an option for AL-CA patients as well (57–59). Cardiac amyloidosis patients have a higher heart transplant waiting list mortality than cardiomyopathy from other causes (51,60). The 2018 UNOS heart allocation scheme gives priority clearance for CTx to patients with CA as they are at high risk of mortality (51,61). For patients with ATTRwt-CA or ATTRv-CA with the V122I mutation, heart transplantation alone is usually sufficient, but may need to be considered for dual heart/liver transplantation in the presence of other variants such as Thr60Ala (52). However, follow-up studies unfortunately show that disease recurrence and improvement in extracardiac manifestations may occur after Tx (62). Cardiac transplantation was performed on a patient with ATTRwt-CA mutation in our Ege University heart transplant program. However, the patient died from Candida sepsis at 6 weeks postoperatively.

## CONCLUSION

Cardiac amyloidosis is one of the causes of restrictive cardiomyopathy. It is characterized by the extracellular accumulation of abnormal proteins that show birefringence in Congo red-polarized light. Regardless of the subtype of the deposited amyloid, the disease results in progressive heart failure if left untreated. Its incidence has increased with the development of diagnostic methods in recent years and the increased sensitivity of clinicians to disease symptoms. This has enabled both the understanding of the pathogenesis of the disease and the development of new treatment options. A growing number of studies on the disease have made significant advances. This situation raises our hope that there may be significant changes in the diagnosis, treatment, and follow-up of cardiac amyloidosis in the coming years.

## Conflict of Interest

The authors declare no potential conflicts of interest regarding the research, authorship and/or publication of this article. Only the authors are responsible for the content and writing of the article.
